# Knockdown of histidine-rich calcium-binding protein (HRC) suppresses liver fibrosis by inhibiting the activation of hepatic stellate cells

**DOI:** 10.1242/bio.019828

**Published:** 2016-11-23

**Authors:** Jingmei Liu, Mengke Li, Jin Gong, Ping Han, Yunwu Wang, Dongxiao Li, Dean Tian, Jiazhi Liao

**Affiliations:** 1 Department of Gastroenterology, Tongji Hospital, Tongji Medical College, Huazhong University of Science and Technology, Wuhan 430030, China; 2 Department of Gastroenterology, Zhoushan Hospital, Zhoushan 316000, China

**Keywords:** Liver fibrosis, Hepatic stellate cells, HRC, ER stress

## Abstract

The histidine-rich calcium-binding protein (HRC) is a regulator of Ca^2+^ homeostasis and it plays a significant role in hepatocellular carcinoma (HCC) progression. However, the relationship between HRC and liver fibrogenesis is still unknown. Our data demonstrates that HRC was upregulated in fibrotic liver and activated hepatic stellate cells (HSCs). TGF-β treatment increased α-SMA and HRC expression dose-dependently in HSCs. Repression of HRC reduced α-SMA, CTGF and collagen expression, and inhibited HSC proliferation and migration. In addition, we found that the anti-fibrosis effect of HRC knockdown was associated with endoplasmic reticulum (ER) stress. Silencing of HRC decreased the expression of ER stress and autophagy markers. Moreover, ER stress agonist thapsigargin (TG) enhanced, whereas ER stress antagonist 4-phenylbutyric acid (4-PBA) alleviated HSCs activation and autophagy. In conclusion, these data indicate that depletion of HRC inhibited HSC activation through the ER stress pathway, and HRC may be a potential regulator of liver fibrosis.

## Abstract (precis)

**Summary:** We provide the first evidence to confirm that HRC is a factor in liver fibrosis, and we clarify the molecular mechanism of HRC in hepatic stellate cell activation and liver fibrosis.

## INTRODUCTION

Liver fibrosis is a wound-healing response to chronic liver injury, and it also considered as a pre-cancerous condition which may result in hepatocellular carcinoma (HCC). Activated hepatic stellate cells (HSCs) are responsible for the excessive deposition of extracellular matrix (ECM) proteins during hepatic fibrogenesis (Friedman, 2004). Prevention or reversal of HSC activation has been claimed as a potential approach to attenuate liver fibrosis (Bataller and Brenner, 2005). Although many potential anti-fibrotic targets have been identified, effective clinical therapies are still lacking. Therefore, it is urgent to develop novel strategies that prevent the progression of liver fibrosis.

The histidine-rich calcium-binding protein (HRC) is a regulator in Ca^2+^ homeostasis (Park et al., 2012). Calcium signals may be of particular importance in HSC biology (Kruglov et al., 2007). Activated HSCs are capable of elevated ECM deposition, increased proliferation and enhanced migration, which were identified as the Ca^2+^-dependent responses (Melton et al., 2006; Soliman et al., 2009). Recently, a number of calcium-binding proteins have been implicated in liver fibrogenesis, such as S100A4, SPARC and annexin A2 (Zhang et al., 2010; Atorrasagasti et al., 2011, 2013). However, the role of HRC in liver fibrosis has not been investigated so far.

Recent data indicate that ER stress plays a pivotal role in the progression of liver diseases, including liver fibrosis (Malhi and Kaufman, 2011; Pagliassotti, 2012). Inhibition of ER stress prevented hepatic fibrosis by attenuating HSC activation (Men et al., 2015). Our previous study showed that HRC promoted the growth of liver cancer by inhibiting ER stress-induced apoptosis (Liu et al., 2015). However, the effect of HRC on ER stress in HSCs remains unknown. It is noteworthy that ER stress induces the activation of HSCs through autophagy, and blockage of ER stress suppresses autophagic activity, thus inhibiting HSCs activation (Hernández-Gea et al., 2013; Men et al., 2015).

For the first time, we discovered that HRC was upregulated in fibrotic liver and activated HSCs. Moreover, we demonstrated that HRC knockdown inhibited the activation, proliferation and migration of HSCs, which was partly attributed to ER stress and autophagy.

## RESULTS

### HRC is upregulated in fibrotic liver

**Fig. 1.**
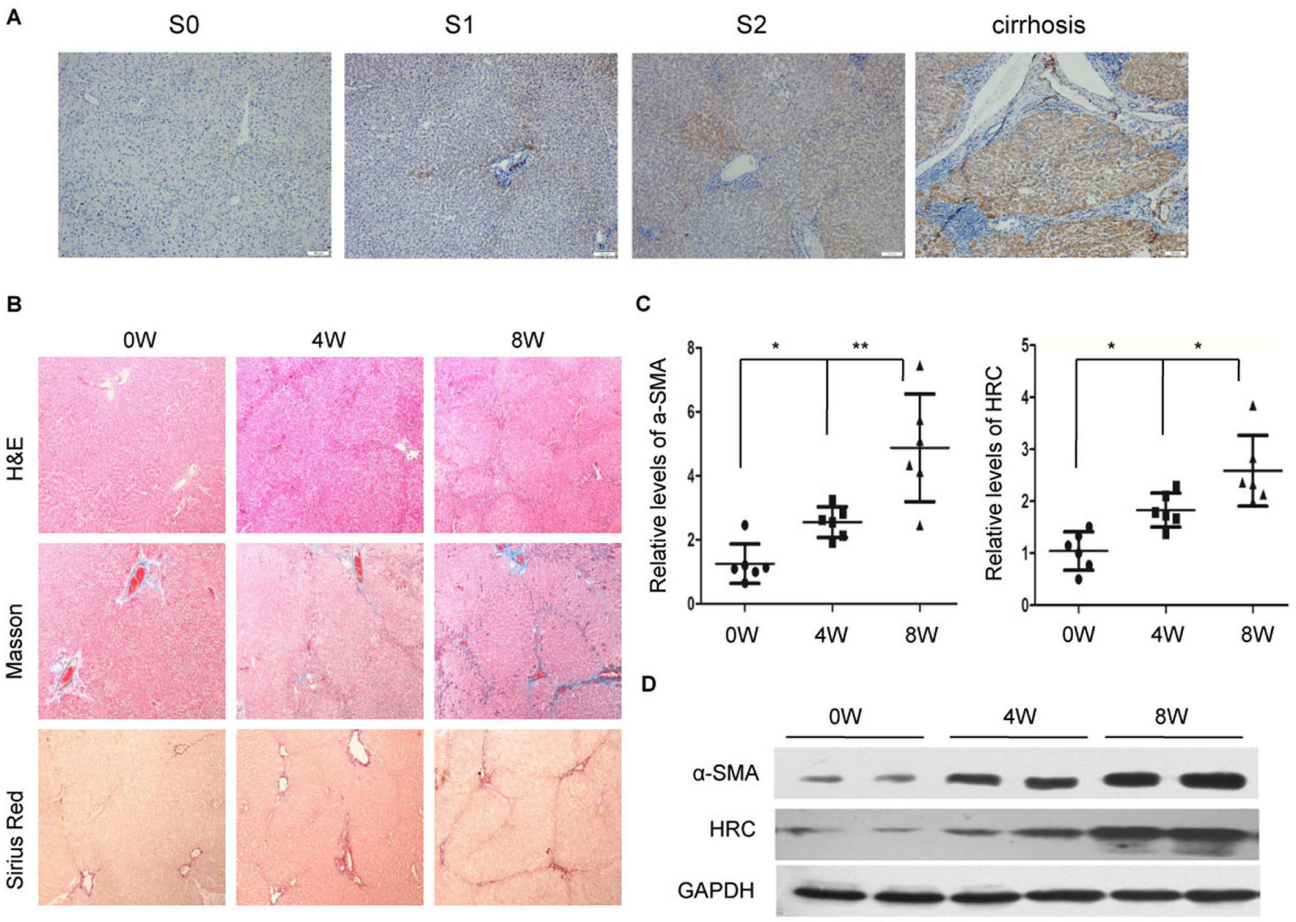
**HRC is upregulated in fibrotic liver.** (A) Representative immunohistochemical staining shows the expression of HRC in human samples. S0 represents no inflammation and no fibrosis of liver, S1 represents inflammation in portal area and peri-sinusoid fibrosis, S2 represents mild inflammation and fibrous septum formation. Scale bars: 100 µm. (B) Representative H&E, Masson and Sirius Red staining (100×) of TAA-induced liver fibrosis in rats. W, weeks. 4W represents rats treated with TAA for 4 weeks, 8W represents rats treated with TAA for 8 weeks. (C) Box plot (25^th^ percentile, mean, 75^th^ percentile) of the mRNA levels of α-SMA and HRC in TAA-induced liver fibrosis in rats measured by RT-qPCR (*n*=6). *P<0.05, **P<0.01. (D) Representative image shows the protein levels of α-SMA and HRC in TAA-induced liver fibrosis in rats. GAPDH was used as a loading control.

To explore the role of HRC in liver fibrosis, immunohistochemistry was performed to determine the expression of HRC in human samples. The results showed that the expression of HRC was gradually increased along with the severity of liver fibrosis, and HRC was significantly overexpressed in cirrhosis (Fig. 1A). In addition, we detected the expression of HRC in a thioacetamide (TAA)-induced liver fibrosis animal model, which is widely used for the study of liver fibrosis (He et al., 2015). As expected, TAA administration led to liver fibrosis as confirmed by H&E, Masson and Sirius Red staining, which showed severe distortion of architecture and collagen deposition (Fig. 1B). Moreover, TAA treatment significantly induced the accumulation of activated HSCs, as indicated by the overexpression of α-smooth muscle actin (α-SMA) (Fig. 1C,D). Moreover, the level of HRC was also upregulated in fibrotic liver (Fig. 1C,D). The obtained results suggest that HRC plays a crucial role in the development of liver fibrosis.

### HRC is overexpressed in activated HSCs

**Fig. 2.**
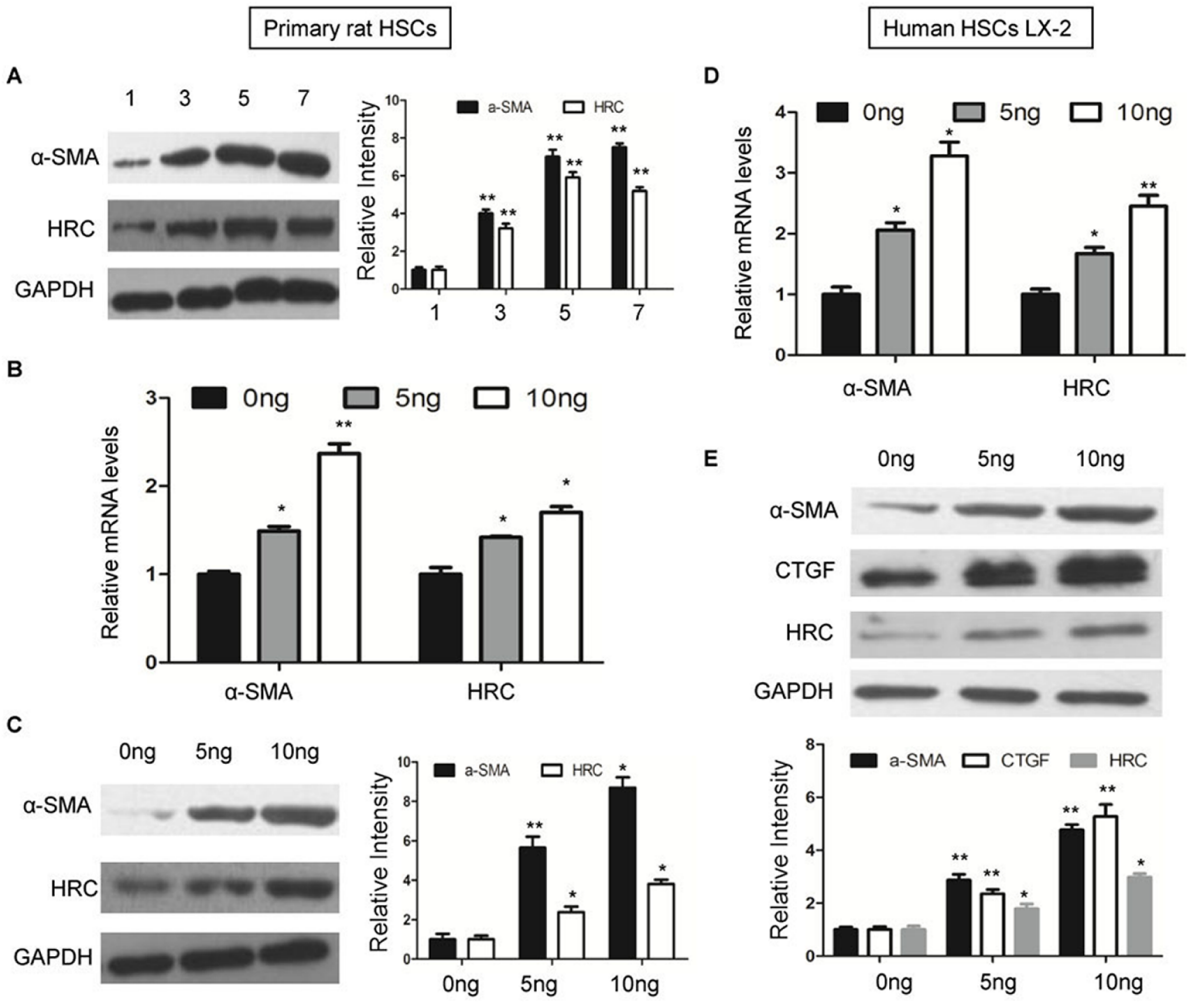
**HRC is over-expressed in activated HSCs.** (A) The expression of α-SMA and HRC were detected by western blot during the spontaneous activation of primary rat HSCs. (B) RT-qPCR analysis of α-SMA and HRC expression by in primary rat HSCs treated with TGF-β. (C) Western blot analysis of α-SMA and HRC expression in primary rat HSCs after TGF-β stimulation. (D) The mRNA levels of α-SMA and HRC in human HSC cell line LX-2 after TGF-β stimulation. (E) Western blot analysis of α-SMA, CTGF and HRC expression in LX-2 cells treated with TGF-β. GAPDH was used as a loading control in all western blots. Error bars represents the mean±s.d. of three separate experiments. *P<0.05, **P<0.01.

Freshly isolated (1 day) or 7-day cultured primary rat HSCs were used as quiescent and activated cells, respectively, as described in our previous study (He et al., 2015).The expression of α-SMA and HRC were increased accompanied with HSCs activation (Fig. 2A). Additionally, primary rat HSCs were stimulated by TGF-β, which is the most potent pro-fibrogenic mediator in activating HSC and stimulating collagen production (Gressner et al., 2002). As shown, TGF-β stimulation induced a higher expression of α-SMA and HRC in a dose-dependent manner (Fig. 2B,C). For a further confirmation, the human HSC cell line LX2 was also stimulated with TGF-β. Similarly, the level of HRC was significantly elevated, in line with the increased α-SMA and CTGF expression (Fig. 2D,E). These data indicate that HRC expression levels increase during HSC activation.

### HRC knockdown inhibits the activation, proliferation and migration of HSCs

**Fig. 3.**
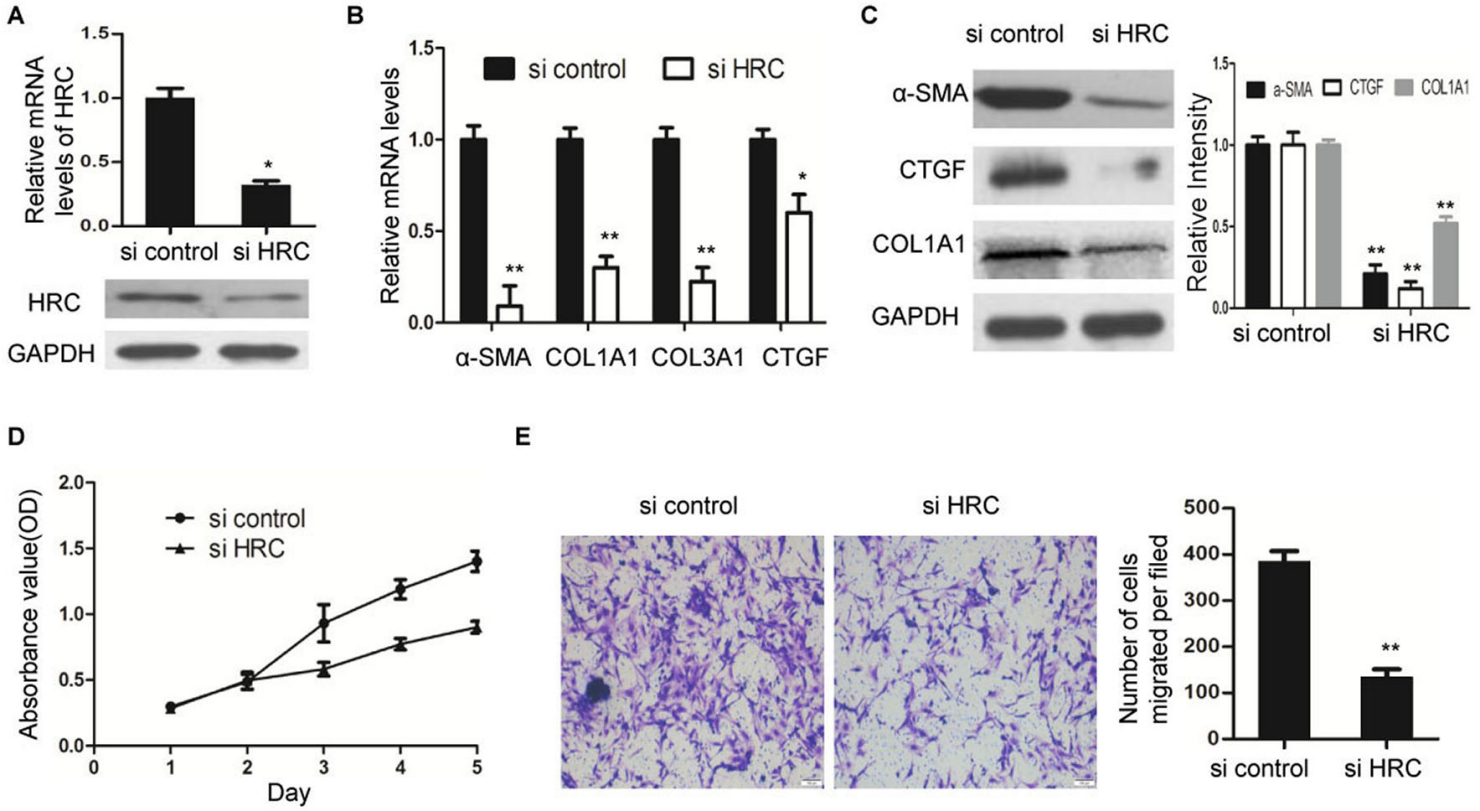
**HRC knockdown inhibits the activation, proliferation and migration of HSCs.** (A) The expression of HRC in LX-2 cells transfected with siRNAs (si control or si HRC) was measured by RT-qPCR. (B) RT-qPCR analysis of fibrogenesis-associated gene expression, including α-SMA, CTGF, COL1A1 and COL3A1. (C) Western blot analysis of α-SMA, CTGF and COL1A1 expression. GAPDH was used as a loading control. (D) The proliferation rates of LX-2 cells transfected with siRNAs (si control or si HRC) were determined by cell-counting assay. (E) Cell migration in LX-2-siHRC and LX-2-sicontrol cells were analyzed by transwell assay. Scale bars: 100 μm. Error bars represent the mean±s.d. of three separate experiments. *P<0.05, **P<0.01.

Next, we wondered whether HSCs activation could be inhibited by HRC knockdown. RNA interference was used to downregulate HRC expression in LX-2 cells (Fig. 3A). Reduction of HRC resulted in a significant decrease in the expression of α-SMA, accompanied with the downregulation of other fibrosis-related genes, including CTGF, COL1A1 and COL3A1 (Fig. 3B,C). Previous research has demonstrated that the proliferation and migration rates of activated HSCs are critical to the progression of liver fibrosis (Brenner et al., 2000). Therefore, we assessed the effect of HRC on HSC proliferation and migration respectively. Compared to the control siRNA, LX-2 cells transfected with siHRC had a worse proliferative capacity (Fig. 3D). In addition, the migration capability of LX-2 cells was also suppressed by HRC knockdown (Fig. 3E). The results illustrate that HRC knockdown attenuates HSC activation, proliferation and migration.

### HRC knockdown inhibits HSCs activation through the ER stress pathway

**Fig. 4.**
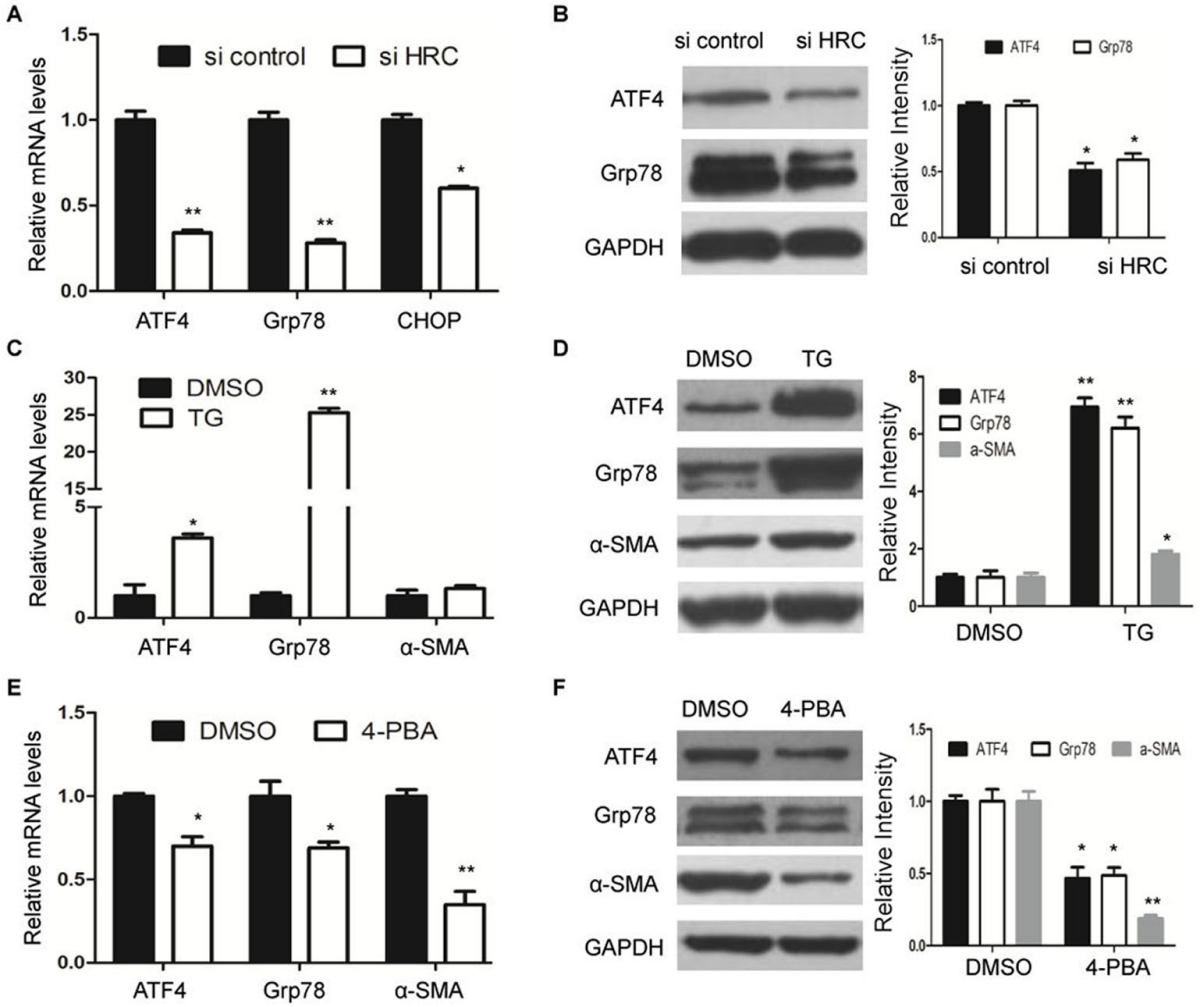
**HRC knockdown inhibits HSC activation through the ER stress pathway.** (A) RT-qPCR analysis of the expression of ER stress molecular indicators, including ATF4, GRP78 and CHOP. (B) The protein levels of ATF4 and Grp78 were determined by western blot. (C) RT-qPCR and (D) western blot analysis of ATF4, Grp78 and α-SMA in LX-2 cells treated with thapsigargin (TG). (E) The mRNA and (F) protein levels of ATF4, Grp78 and α-SMA in LX-2 cells treated with 4-phenylbutyric acid (4-PBA). GAPDH was used as a loading control. Error bars represents the mean±s.d. of three separate experiments. *P<0.05, **P<0.01.

As endoplasmic reticulum (ER) stress is emerging as a well-described determinant of HSCs activation (Tanjore et al., 2013), we hypothesized that HRC knockdown might inhibit ER stress. In accordance with our expectation, the expression of ER stress molecular indicators, such as ATF4, GRP78 and CHOP, were significantly decreased in HRC-knockdown LX-2 cells (Fig. 4A,B). Furthermore, thapsigargin (TG), the classical ER stress inducer, not only triggered ER stress (Fig. 4C,D), but induced HSCs activation, as shown by a strong increase in the protein level of α-SMA (Fig. 4D). Conversely, 4-phenylbutyric acid (4-PBA), an ER stress modulator, led to the downregulation of α-SMA (Fig. 4E,F). These results suggest that repression of HRC inhibited HSC activation partly through the ER stress pathway.

### HRC knockdown inhibits autophagy in HSCs

**Fig. 5.**
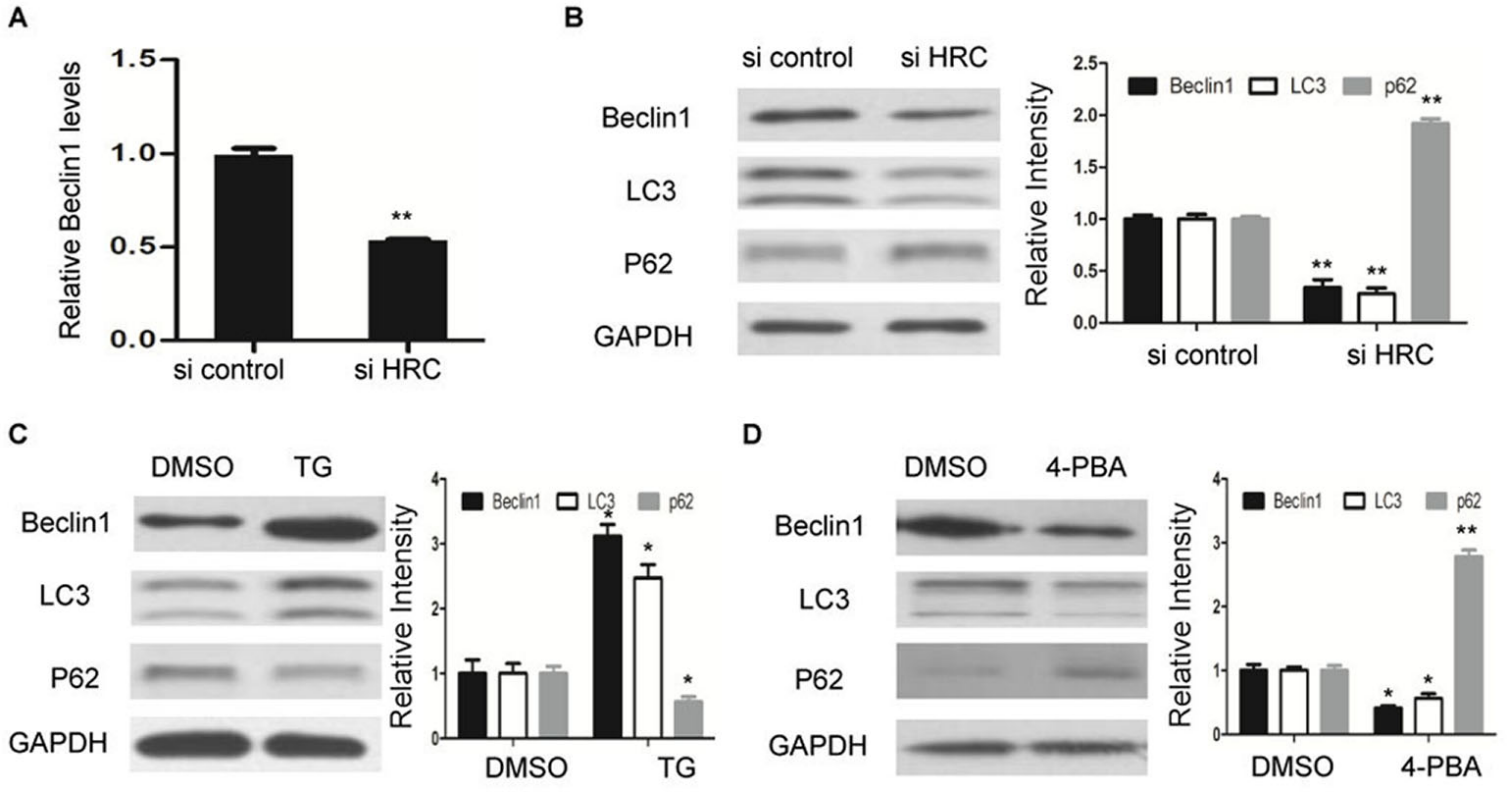
**HRC knockdown inhibits autophagy in HSCs.** (A) RT-qPCR analysis of Beclin-1 expression in LX-2 cells transfected with siRNAs (si control or si HRC). (B) The protein levels of autophagic markers, Beclin1, LC3 and p62, were determined by western blot. (C) Western blot analysis of Beclin1, LC3 and p62 expression in LX-2 cells treated with TG. (D) The expression of Beclin1, LC3 and p62 in LX-2 cells after 4-PBA treatment were determined by western blot. GAPDH was used as a loading control. Error bars represents the mean± s.d. of three separate experiments. *P<0.05, **P<0.01.

Recent data has indicated that ER stress induces HSC activation through autophagy (Hernández-Gea et al., 2013). Therefore, we evaluated the effect of HRC on autophagy. Interestingly, HRC knockdown reduced autophagic activity, as shown by the decreased levels of Beclin-1 and LC3, and the increased level of p62 (Fig. 5A,B). In addition, we also demonstrated that ER stress inducer TG increased the levels of Beclin-1 and LC3, and decreased p62 expression (Fig. 5C). However, the levels of Beclin-1 and LC3 were markedly decreased by 4-PBA, which is identified as an ER stress antagonist. Moreover, 4-PBA treatment induced the expression of p62 (Fig. 5D). Taken together, these results demonstrate that repression of ER stress caused by HRC knockdown leads to the inhibition of autophagy, which eventually suppresses HSCs activation.

## DISCUSSION

The histidine-rich calcium-binding protein (HRC) participates in the regulation of Ca^2+^ homeostasis. Increasing evidence has revealed that Ca^2+^ signaling plays a crucial role in HSC activation, proliferation and contraction (Melton et al., 2006; Soliman et al., 2009). Furthermore, a variety of calcium-binding proteins are involved in the development of liver fibrosis (Zhang et al., 2010; Atorrasagasti et al., 2013; Chen et al., 2015). Chen et al*.* reported that S100A4 promoted liver fibrosis by activating HSCs (Chen et al., 2015), and Atorrasagasti et al*.* found that down-regulation of SPARC in activated HSCs exerts an anti-fibrotic effect (Atorrasagasti et al., 2011). However, the biological function of HRC in liver fibrosis remains unknown.

Previous literature has demonstrated that HRC plays an important role in cardiac fibrosis, and our data support this notion in liver fibrosis as well. Here, for the first time, we found that HRC was significantly upregulated in fibrotic liver, and during the activation of HSCs, the expression of HRC could be obviously induced. Furthermore, we demonstrated that HRC knockdown inhibited HSCs activation, proliferation and migration *in vitro*. Mechanistic studies revealed that ER stress and autophagy were responsible for the observed effects of HRC on HSC activation. Collectively, these results suggest that HRC may be a promising target for anti-fibrotic therapy.

The central step in liver fibrosis is the activation of HSCs, inhibition of HSC activation is considered a key target for the treatment of hepatic fibrosis (Friedman, 2004). To assess whether HRC is an important factor involved in HSC activation, RNA interference (RNAi) was used to knock down HRC expression in HSCs. Knockdown of HRC reduced the expression of α-SMA, along with other genes relative to liver fibrosis, such as Col1A1, Col3A1 and CTGF. Enhanced proliferation and migration of HSCs lead to collagen deposition, and finally resulted in the development of fibrotic scar (Brenner et al., 2000). We next investigated the functional role of HRC in HSC proliferation and migration. Within our expectations, the proliferation activity and migration ability of HSCs were obviously suppressed by HRC knockdown.

Accumulating evidence suggests that ER stress plays essential roles in the progression of tissue fibrosis, including in liver, lung and kidney (Korfei et al., 2008; Malhi and Kaufman, 2011; Taniguchi and Yoshida, 2015). Aberrant ER stress signaling has been reported in liver fibrosis. ER stress pathway components Grp78 and CHOP were markedly increased in experimentally induced liver fibrosis in transgenic mice (Mencin et al., 2007). Tamaki et al*.* demonstrated that liver fibrosis was greatly attenuated in CHOP-deficient mice following bile duct ligation (Tamaki et al., 2008). Our data demonstrated that HRC knockdown decreased the expression of ER stress markers, including Grp78, ATF4 and CHOP. Moreover, we also confirmed that the ER stress inducer TG enhanced, while the ER stress modulator 4-PBA reduced, the expression of α-SMA. These findings indicated that silencing of HRC inhibited HSC activation by the ER stress pathway.

Recent research has suggested that autophagic flux increases during HSC activation, and the impact of ER stress on HSC activation is partly through autophagy (Hernández-Gea et al., 2012). We further analyzed the effect of HRC knockdown on Beclin-1, LC3 and p62 expression, which represent the extent of autophagy. The levels of Beclin-1 and LC3 were significantly downregulated, and the level of p62 was increased by HRC knockdown, which is consistent with our expectation. In addition, we also detected autophagic makers in response to ER stress. Consistent with a previous study (Men et al., 2015), ER stress stimuli led to elevated Beclin1 and LC3 expression, and the level of p62 was slightly decreased by TG, while a lower ER stress level inhibited autophagic activity. These results suggested that the reduction of ER stress induced by HRC knockdown led to the inhibition of autophagy, thus suppressing HSCs activation.

In conclusion, our study demonstrated that knockdown of HRC inhibit HSC activation and liver fibrosis *in vitro*, and further studies should be carried out to clarify the role of HRC in liver fibrosis *in vivo*.

## MATERIALS AND METHODS

### Human samples

Human samples (*n*=45) were divided into five groups according to the severity of liver fibrosis, including S0 (*n*=5), S1 (*n*=10), S2 (*n*=10), S3 (*n*=2) and S4 (*n*=18), which were diagnosed by the pathologists at Tongji Hospital of Tongji Medical College, Huazhong University of Science and Technology. All procedures involving human participants were performed after obtained their informed consent, which was also in accordance with the Medical Ethics Committee of Tongji Hospital.

### Animal models of liver fibrosis

Male Sprague–Dawley (SD) rats weighting about 200 g were randomly divided into three groups (*n*=6/group). Rats were treated with thioacetamide (TAA) or saline (200 mg kg^−1^) by intraperitoneal (i.p.) injection twice a week for 8 weeks. All procedures performed in studies involving animals were approved by the Institutional Laboratory Animal Care and Use Committee by the Institutional Laboratory Animal Care and Use Committee of Tongji hospital.

### Primary HSC isolation and culture

Rat primary HSCs were isolated from livers by *in situ* perfusion with pronase and collagenase and single-step Nycodenz gradient centrifugation as described previously (Kawada et al., 1998; Weiskirchen and Gressner, 2005).

### Immunohistochemistry

Histological analysis of fibrosis was performed on fixed liver tissue, embedded in paraffin, and sectioned at a thickness of 4 μm. The 4 μm thick sections were used for Hematoxylin and Eosin (H&E), Masson and Sirius Red staining. An HRC polyclonal antibody (Abonva, CA, USA) was used to detect the expression of HRC in fibrotic liver.

### RNA extraction and real-time RT-PCR

Total RNA was extracted using TRIzol reagent (Invitrogen, CA, USA). Reverse-transcribed complementary DNA was synthesized using the PrimeScript RT reagent kit (TaKaRa, Otsu, Japan). Real-time polymerase chain reaction was performed using SYBR Premix ExTaq (TaKaRa) on an ABI StepOne Real-Time PCR System (Applied Biosystem, CA, USA). The sequences of the primers used for PCR are listed in Table S1.

### Western blot

Western blot was performed as previously described (Han et al., 2014). Briefly, samples containing 30 μg of total protein were resolved on 10% polyacrylamide SDS gels and electrophoretically transferred to polyvinylidene difluoride (PVDF) membranes. The membranes were blocked with 5% skim milk, incubated with appropriate primary antibodies and HRP-conjugated suitable secondary antibodies, followed by detection with enhanced chemiluminescence reagents (Pierce Chemical, IL, USA). GAPDH was used as a loading control. The antibodies are listed in Table S2.

### RNA interference

For RNA interference, HRC siRNA was synthesised by RiboBio (Guangzhou, China), and then transfected into LX-2 cells using lipofectamine 2000 (Invitrogen) according to the manufacturer's instructions. The sequence of siHRC was designed as follows: CCACAGAGACGAGGAAGAA.

### CCK-8 assay

Cell proliferation was analyzed by Cell Counting Kit-8 (CCK-8) assay (Dojindo Laboratories, Kumamoto, Japan) according to the manufacturer's instructions.

### Transwell migration assay

Cell migration assay was performed using transwell chambers (6.5 mm diameter and 8 µm pore size; Costar; Corning Inc.). Briefly, a total of 5×10^4^ cells in 0.2 ml media were plated in the upper chambers, 600 μl DMEM medium containing 10% fetal calf serum (Invitrogen Gibco, CA, USA) was added to the lower chamber. After 24 h, cells that migrated through the membrane to the lower surface were fixed with 4% paraformaldehyde, stained with crystal violet and counted by microscopy (Olympus, NY, USA).

### Statistical analysis

Data are presented as means±s.d. Student's *t*-test was performed to assess the significance of differences between two groups. A *P*-value <0.05 was considered statistically significant.
